# Associations between air pollution and hospitalization for cardiovascular disease: a time series study in Nanchong

**DOI:** 10.3389/fpubh.2025.1504411

**Published:** 2025-03-07

**Authors:** Zaiyong Zheng, Yanman Li, Qinglu Jiang, Fangfang Zang, Yang Yu, Rongchuan Yue, Houxiang Hu, Chunxiang Zhang

**Affiliations:** ^1^Key Laboratory of Medical Electrophysiology, Ministry of Education, Department of Cardiology, Luzhou, China; ^2^Basic Medicine Research Innovation Center for Cardiometabolic Diseases, Ministry of Education, Luzhou, China; ^3^The Affiliated Hospital of Southwest Medical University, Southwest Medical University, Luzhou, China; ^4^Department of Cardiology, Academician Workstation, The Affiliated Hospital of North Sichuan Medical College, North Sichuan Medical College, Nanchong, China

**Keywords:** air pollutants, cardiovascular disease, environmental epidemiology, human health, cardiovascular disease hospitalization

## Abstract

**Objective:**

To investigate the acute effects of air pollution on the daily hospitalizations for cardiovascular disease.

**Methods:**

Data of daily hospitalization for cardiovascular disease were collected from the hospital electronic health record system in Nanchong. The air pollutants and meteorological data were obtained from the fixed monitoring stations. We performed over-dispersed Poisson regression incorporated with distributed lag models to assess associations between short-term exposure to air pollutants and the risk of cardiovascular disease hospitalizations.

**Results:**

A total of 373,390 hospitalizations for cardiovascular diseases were identified. We found that a 10 μg/m^3^ increase in 7-day average concentrations of PM_2.5_ and PM_10_ was associated with 1.15% (95%CI: 0.55–1.76%) and 0.51% (95%CI: 0.19–0.82%) higher cardiovascular disease admissions. NO_2_ presents the largest adverse effect. The risk of cardiovascular disease admission increased by 6.26% with per 10 μg/m^3^ increase in NO_2_ for lag07.

**Conclusion:**

Short-term exposures to high concentrations of air pollutants increased the risk of hospitalization for cardiovascular disease. Policymakers need to develop policies and strategic plans to combat air pollution.

## 1 Introduction

Air pollution has been a global public concern. According to the World Health Organization (WHO), more than 99% of the global population lives in area with air pollution (1). In 2021, particulate matter air pollution was identified as the leading factor contributing to the global disease burden (2), accounting for 6.7 million premature deaths annually on a worldwide scale (3). Approximately 4.2 million deaths are attributed to ambient air pollution, with 25% of these fatalities linked to ischemic heart disease (4). In China, the mortality rate from CVD due to ambient particulate matter pollution has shown a consistent increase from 1990 to 2019 (5). Reports from the National Center for Cardiovascular Disease of China indicated that in 2019, more than 1.42 million excess deaths were associated with ambient particulate matter pollution (3). The high exposure risks and limited regenerative capability make the cardiovascular system become vulnerable to the hazardous effects of air pollution. Exposure to air pollution, such as particulate matters (PM_10_ and PM_2.5_), SO_2_, NO_2_, O_3_ and CO, has been associated with a significant increase in the risk of CVD (1). A nationwide cohort study conducted in China demonstrated that each 10 μg/m^3^ increment in PM_2.5_ exposure, corresponded to a 25.1% increase in CVD and a 16.4% rise in CVD mortality (6). Recent empirical evidence also indicates that maternal exposure to air pollution is associated with an elevated risk of congenital heart defects in newborns. A nationwide study of 1,434,998 births found a 2% increase in risk of congenital heart defects for every 10 μg/m^3^ rise in PM_2.5_ exposure (7, 8). These results underscore the significant hazards that air pollution poses to CVD. Therefore, investigating the impact of air pollution on cardiovascular health is important. Given the vast geographical span of China, air quality exhibits considerable variation across different regions. It is crucial to analyze localized data and evaluate the detrimental effects of air pollutants in specific areas. Moreover, the relationship between air pollution and CVD has not been previously documented in Nanchong. In this study, we utilized hospital medical records from Nanchong to assess the influence of air pollutants on hospital admissions related to cardiovascular disease.

## 2 Methods

### 2.1 Data collection

Data of hospitalization admission for cardiovascular disease, encompassing both initial and recurrent cases, were retrieved from the electronic health record system of the affiliated hospital of North Sichuan Medical College from June 1, 2015, to December 31, 2023. Patients with discharge diagnoses containing cardiovascular disease according to ICD-10 (ICD-10: I00-I99) were included. I00-I99 contains: I00-I02: Acute rheumatic fever; I05-I09: Chronic rheumatic heart diseases; I10-I15: Hypertensive diseases; I20-I25: Ischemic heart diseases; I26-I28: Pulmonary heart disease and diseases of pulmonary circulation; I30-I52:Other forms of heart disease; I60-I69: Cerebrovascular diseases; I70-I79: Diseases of arteries, arterioles and capillaries; I80-I89: Diseases of veins, lymphatic vessels and lymph nodes, not elsewhere classified; I95-I99: Other and unspecified disorders of the circulatory system.

**Figure 1:**
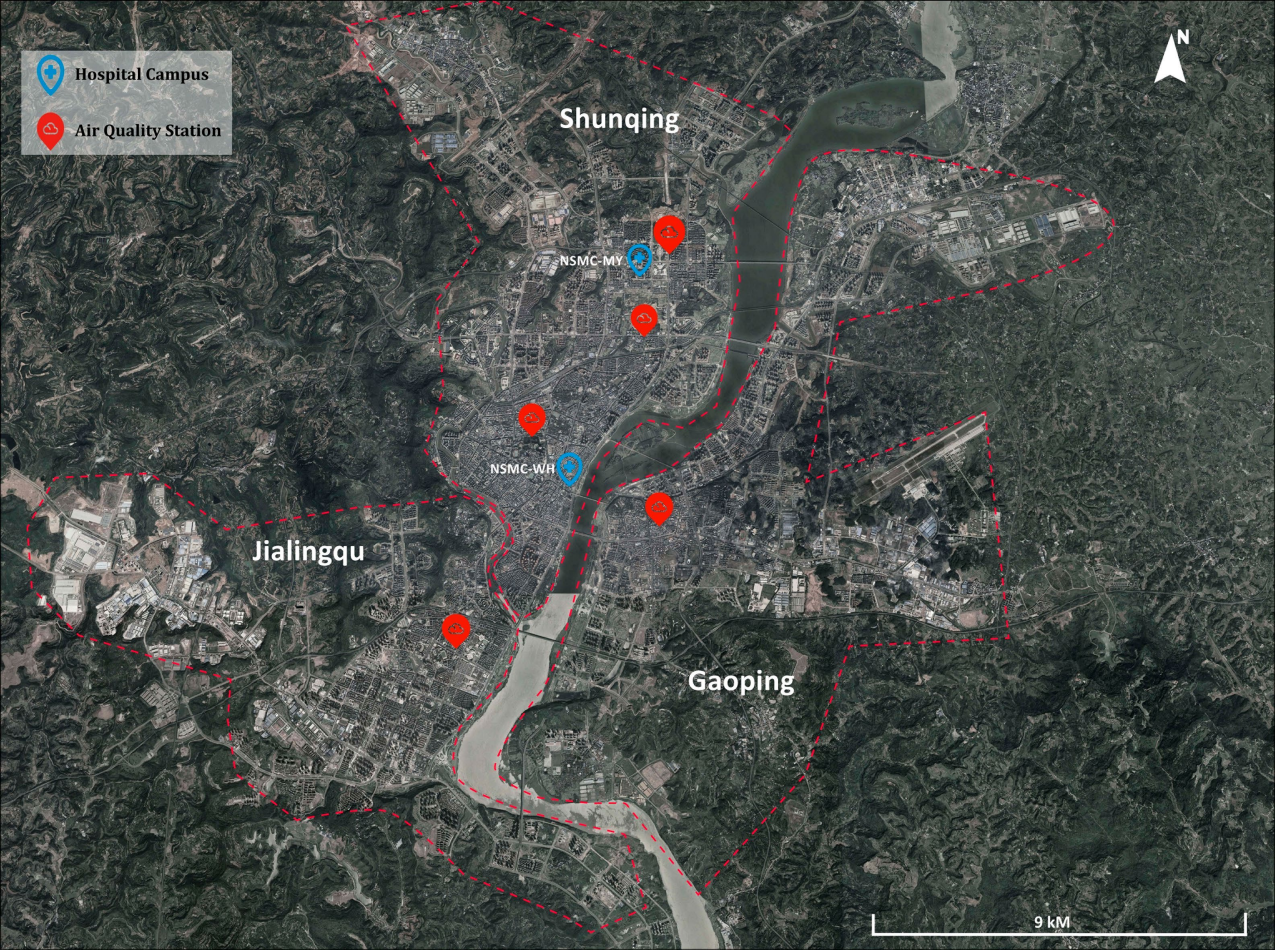
Distribution of air quality monitoring stations and hospital locations in Nanchong.

Nanchong is situated in the southwest region of China, and due to the barrier of the Qinling mountains, Nanchong avoids impacts of dust storm from the Northwest China. The urban area of Nanchong consists of the Shunqing, Gaoping, and Jialing districts. Covering an area of 2,637 square kilometers, the urban area of Nanchong is home to 2.101 million inhabitants. There are four air quality monitoring stations located in the urban area of Nanchong (Figure 1). Air quality data for each patient were matched with the nearest station. As no further detail can be provided, patients from the Shunqing district had their data averaged from the three stations within the district’s jurisdiction: LianYouChang station, Shiwei Jiance station and ShiWei station. Since some patients registered their address as Shixiaqu, an old name for the urban area of Nanchong, the air pollutant data for Shixiaqu was matched with the average data for the entire urban area of Nanchong. Additionally, on May 10, 2021, the Affiliated Hospital of North Sichuan Medical College moved from its former location at the southern part (Affiliated Hospital of NSMC-WH campus) to the northern part (Affiliated Hospital of NSMC-MY campus) of Shunqing district.

We collected daily data on air pollutants and climate factors from the PM_2.5_ historical database.^1^ The air pollutants include PM_2.5_, PM_10_, CO, SO_2_, NO_2_, and O_3_, while climate factors encompass temperature, wind, relative humidity and precipitation. The exposure levels of air pollution for each patient were matched from the monitoring station closet to their home address. The study was approved by the Human Ethics Committee of the Affiliated Hospital of North Sichuan Medical College (NUMBER). Informed consent was not specifically required since personal identifiers were not involved.

### 2.2 Statistical analysis

Spearman correlation coefficients was calculated to explore the relationship between air pollutants and meteorological factors. As the daily admission followed an overdispersion Poisson distribution, we applied quasi-Poisson regression models to estimate the impact of short-term exposure of air pollutants on daily hospitalizations for cardiovascular disease. Air pollutants included PM_2.5_, PM_10_, SO_2_, O_3_, NO_2_, and CO, while meteorological factors comprised daily average temperature (Ave.Temp.), wind speed, relative humidity, and precipitation were included in the regression model.


(1) $$\begin{array}{l} \log\left[E\left(Yt\right)\right]=\alpha +\beta \times Z_{t}+\mathrm{DOW}+\mathrm{ns}(\mathrm{Ave}.\mathrm{Temp}.,\mathrm{df}\\ =3+\mathrm{ns}\left(\mathrm{Relative Humidity,df}=3\right)\\ +\mathrm{ns}\left(\mathrm{Times,df}=3*\mathrm{years}\right) \end{array}$$

In Equation 1, Y*t* represents the daily hospitalization count for circulatory diseases on day t, E(Yt) denotes the expected daily number of cardiovascular disease hospitalizations on day t, *α* represents the intercept, *β* stands for the regression coefficient, $Z_{t}\;$ represents the air pollutants, ns() refers to a nonlinear spline function, and df indicates the degree of freedom (9–11).

The excess risk (ER) of each air pollutant was estimated using the relative risk calculated from Equation 2. The ER of cardiovascular disease hospitalizations for every 10-unit increase in air pollutants was calculated as follows:


(2) ER%=exp(β∗10)−1∗100%

In Equation 2, *β* represents the exposure-response coefficient obtained from quasi-Poisson regression, indicating the association between cardiovascular disease hospitalization and air pollutants. This coefficient reflects the change in the risk of cardiovascular disease hospitalizations for each unit increase in air pollutants.

*p* < 0.05 was considered statistically significant. All statistical analyses were conducted in R software (version 4.1.2). The effects are described as the percent changes and 95% CI in daily count on admissions for CVD per 10 μg/m^3^.

## 3 Results

### 3.1 Descriptive statistics

Table 1 presents the characteristic of participants stratified by gender. A total of 373,390 hospitalization records of cardiovascular disease were documented from June 1, 2015, to December 31, 2023. Among these records, the male-to-female ratio was 1.27:1 (209,233 males vs. 164,157 females), with males accounting for 56.04%. The majority of patients were over 70 years old (41.93%). The most common comorbidity observed were hypertension (169,061 cases, 45.28%), followed by diabetes (86,864 cases, 23.26%), cerebral infarction (82,441 cases, 22.08%), coronary heart disease (68,706 cases, 18.40%) and electrolyte imbalance (63,506 cases, 17.01%).

**Table 1:** Summary statistics of hospitalization records of cardiovascular disease from June 1, 2015, to December 31, 2023.

Characteristic	Female, *N* = 164,157	Male, *N* = 209,9,233	*p*-value
Distinct			0.10
Gaoping	30,066 (18%)	38,744 (19%)	
Jialing	30,177 (18%)	38,481 (18%)	
Shixiaqu	5,755 (3.5%)	7,553 (3.6%)	
Shunqing	98,159 (60%)	124,455 (59%)	
Age			<0.001
0–50	25,375 (15%)	35,159 (17%)	
50–70	69,594 (42%)	86,704 (41%)	
70–80	46,122 (28%)	56,840 (27%)	
> 80	23,066 (14%)	30,530 (15%)	
Number of hospitalizations			<0.001
1	72,544 (49%)	87,825 (48%)	
2	28,262 (19%)	34,038 (18%)	
3	13,703 (9.3%)	17,670 (9.6%)	
4	7,794 (5.3%)	10,811 (5.9%)	
5	5,332 (3.6%)	7,432 (4.0%)	
> 5	19,023 (13%)	26,525 (14%)	
Unknown	17,499 (10.7%)	24,932 (1.2%)	
Comorbidity			
Diabetes	39,068 (27%)	47,796 (26%)	<0.001
Hypertension	75,894 (52%)	93,167 (51%)	<0.001
Coronary Heart Disease	29,450 (20%)	39,256 (21%)	<0.001
Cerebral infarction	35,272 (24%)	47,169 (26%)	<0.001
Rheumatic Heart Diseases	2,825 (1.9%)	1,289 (0.7%)	<0.001
Hyperlipidemia	11,643 (8.0%)	11,923 (6.5%)	<0.001
Hypoalbuminemia	11,528 (7.9%)	16,788 (9.1%)	<0.001
Heart Failure	10,145 (7.0%)	11,348 (6.2%)	<0.001
Arrhythmia	16,775 (12%)	19,916 (11%)	<0.001
Respiratory Infection	33,442 (23%)	47,673 (26%)	<0.001
Asthma	16,775 (12%)	19,916 (11%)	<0.001
Electrolyte Imbalance	30,103 (21%)	33,403 (18%)	<0.001
COPD	9,998 (6.9%)	22,456 (12%)	<0.001
Tumor	20,367 (14%)	33,668 (18%)	<0.001

COPD, Chronic Obstructive Pulmonary Disease.

### 3.2 Air pollution and meteorological data

Table 2 presents the distributions of meteorological parameters and air pollutants. The daily average temperature was 17.88°C, with a daily maximum temperature of 42.80°C and a minimum daily temperature of −2.9°C.

**Table 2:** Baseline data for meteorological parameters and air pollutants in Nanchong, June 1, 2015, to December 31, 2023.

Characteristic	Spring	Summer	Autumn	Winter
Days	828	920	910	843
Temperature (°C)				
Min.	4.40	16.10	3.90	−2.90
Mean	18.23	27.01	17.88	8.78
Max.	35.60	42.80	38.00	24.30
Wind speed (m/s)				
Min.	0.50	0.60	0.40	0.40
Mean	2.00	1.84	1.71	1.63
Max.	7.00	5.90	6.40	5.80
Relative humidity (%)				
Min.	15.00	28.00	25.00	30.00
Mean	54.28	76.70	66.66	67.40
Max.	95.00	100.00	100.00	94.00
Precipitation (mm)				
Min.	0.00	0.00	0.00	0.00
Mean	2.72	5.77	3.11	0.67
Max.	78.10	219.30	93.10	29.20
Air quality (day)				
Mild pollution	66 (8.0%)	41 (4.5%)	60 (6.6%)	245 (29%)
Moderate pollution	0	0	1 (0.1%)	64 (7.6%)
Heavy pollution	0	0	0	18 (2.1%)
Severe pollution	0	0	0	2 (0.2%)
O_3_ (μg/m^3^)				
Min.	18.00	26.00	7.00	10.00
Mean	78.57	92.34	56.21	45.37
Max.	207.83	225.86	170.00	142.86
PM_2.5_ (μg/m^3^)				
Min.	5.00	6.00	5.00	10.00
Mean	41.07	27.46	36.78	70.25
Max.	129.03	93.78	120.64	250.48
PM_10_ (μg/m^3^)				
Min.	8.75	7.36	8.13	11.12
Mean	64.21	44.62	51.71	91.74
Max.	204.57	163.60	181.08	331.50
AQI				
Min.	18.13	18.75	10.63	13.86
Mean	65.61	58.30	54.52	89.47
Max.	173.00	161.14	159.50	300.50
SO_2_ (μg/m^3^)				
Min.	2.13	2.38	1.38	3.75
Mean	9.23	9.54	8.72	9.82
Max.	43.81	35.22	49.73	60.75
NO_2_ (μg/m^3^)				
Min.	6.00	4.88	4.75	5.75
Mean	24.17	18.82	24.16	28.61
Max.	63.72	42.35	70.61	72.70
CO (mg/m^3^)				
Min.	0.23	0.20	0.18	0.18
Mean	0.66	0.54	0.60	0.86
Max.	1.78	1.06	1.25	2.00

During the study period, the daily average concentrations of air pollutants were as follows: PM_2.5_ (42.39 ± 28.68 μg/m^3^), PM_10_ (63.70 ± 40.21 μg/m^3^), SO_2_ (9.45 ± 6.94 μg/m^3^), NO_2_ (24.18 ± 11.09 μg/m^3^), CO (0.67 ± 0.27 mg/m^3^), and O_3_ (68.77 ± 35.97 μg/m^3^). Among different seasons, winter exhibits the highest concentrations of air pollutants such as PM_2.5_, PM_10_, AQI, SO_2_, NO_2_, and CO, while spring demonstrates the highest levels of O_3_, with winter conversely showcasing the lowest O_3_ levels. According to the Technical Regulation on Ambient Air Quality Index (on trial) (HJ 633—2012) of China, air quality is categorized as mild pollution for an AQI range of 101–150, moderate pollution for an AQI range of 151–200, heavy pollution for an AQI range of 201–300, and severe pollution for an AQI exceeding 300. Nanchong experiences the highest number of pollution days during winter, with 2 days (0.2%) of severe pollution heavy pollution, 18 days (2.1%) of moderate pollution, 64 days (7.6%) of moderate pollution, and 245 days (29%) of mild pollution.

### 3.3 The relationships between meteorological parameters and air pollutants

The Spearman correlation analysis was showed in Supplementary Table S1, daily average temperatures are negatively correlated with PM_2.5_ (*r* = −0.352), PM_10_ (*r* = −0.295), NO_2_ (*r* = −0.271), and CO (*r* = −0.336), while positively correlated with O_3_ (*r* = 0.592). Strong correlations are observed between air pollutants, particularly between PM_2.5_ and PM_10_ (*r* = 0.963). However, in contrast to areas affected by dust storms, wind speeds exhibit a negative relationship with PM_2.5_(*r* = −0.346) and PM_10_ (*r* = −0.311) (12).

**Figure 2:**
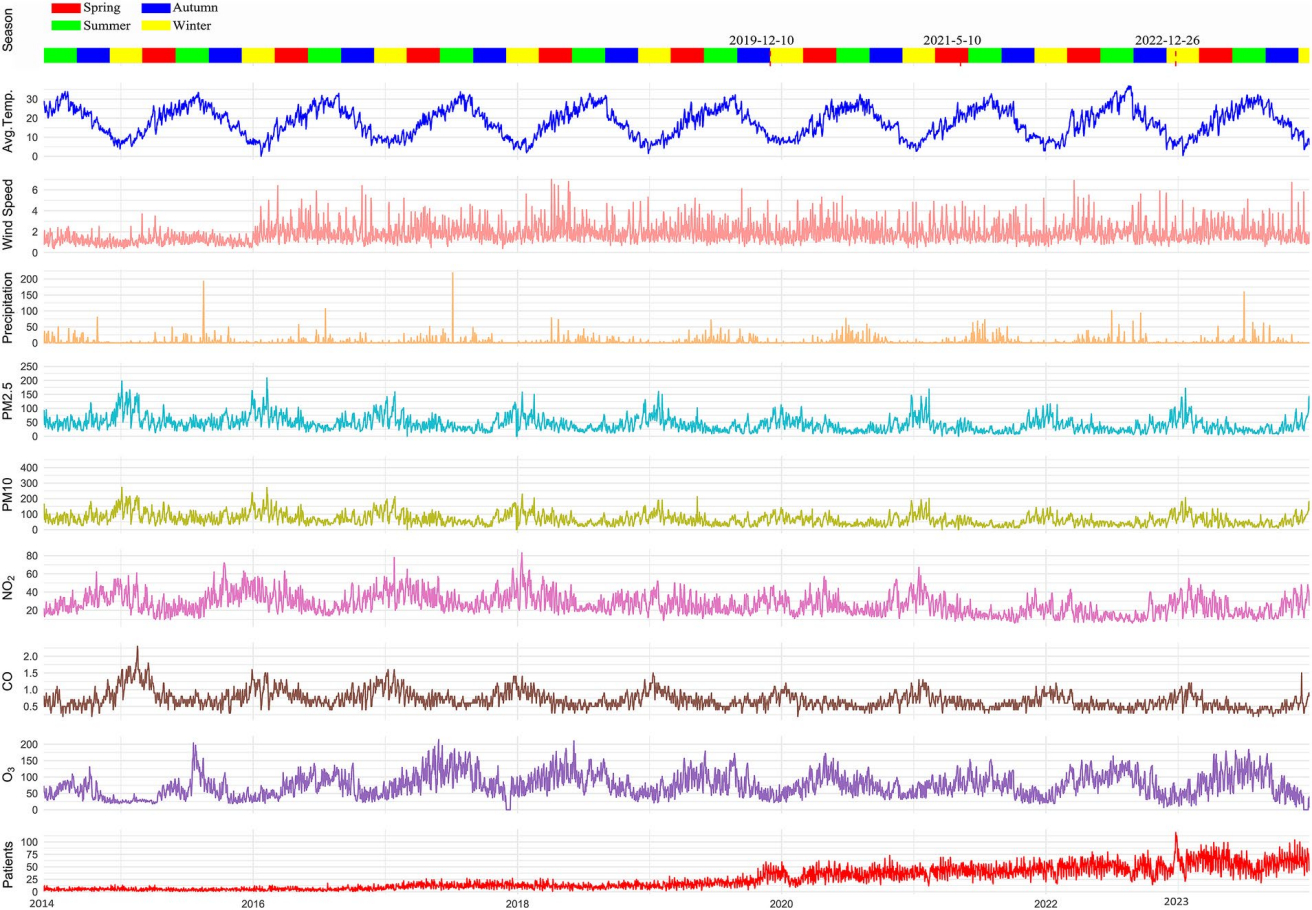
Local meteorological parameters and air pollutants in Nanchong from 2014 to 2023. During the COVID-19 pandemic period from December 10, 2019, to December 26, 2022, the Affiliated Hospital of North Sichuan Medical College relocated to a new campus on May 10, 2021.

Figure 2 represents meteorological parameters, air pollutants, and the number of cardiovascular disease patients from June 1, 2015, to December 31, 2023. In Nanchong, there was significant precipitation and higher temperatures in summer, as well as stronger winds in spring. Additionally, air pollutants have been decreasing since 2018.

### 3.4 Association air pollution and cardiovascular disease hospitalizations

**Figure 3:**
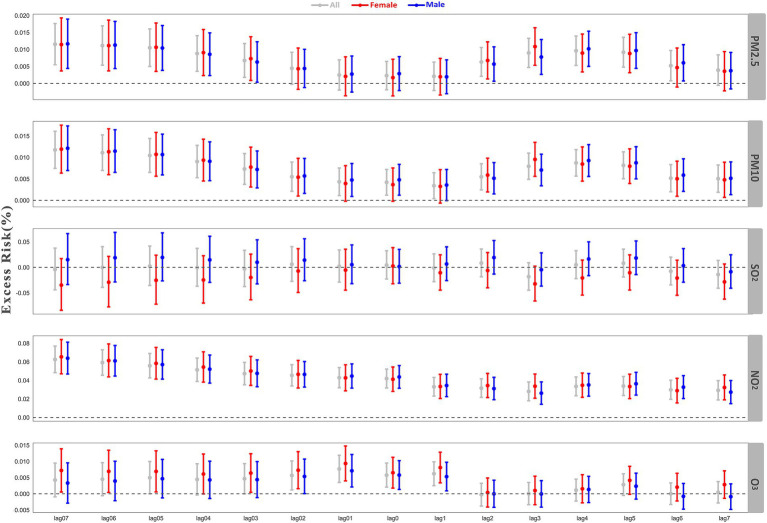
Excess risk (95% CI) of hospitalization for cardiovascular disease associated with an increase of 10 units of pollutant at different lag days using single-pollutant models in Nanchong, June 1, 2015, to December 31, 2023.

The lag effects of air pollutants on the hospitalization of cardiovascular disease were displayed in Figure.3. The concentration of PM_2.5_ showed significant cumulative effect (lag07-lag03) and lag effects (lag2-lag5) on cardiovascular disease patients. The number of cardiovascular disease hospitalization increased by 1.15% (95%CI: 0.55–1.76%) for every 10 μg/m^3^ increase in the average concentration of PM_2.5_ for previous 7 days (lag07). At the same time, PM_2.5_ showed a significant lag effect from lag2 to lag6. As shown in the Figure 3, PM_10_ showed a stronger cumulative and lag effect. Among all patients, both the average level of PM_10_ over the previous 7 days (lag01-lag07) and each individual day’s level over the same period (lag1-lag7) increase the risk of cardiovascular disease hospitalization. The number of cardiovascular disease hospitalization increased by 1.17% (95%CI: 0.74–1.61%) for every 10 μg/m^3^ increase in the average concentration of PM_10_ over the previous 7 days (lag07), and by 0.51% (95%CI: 0.19–0.82%) for every 10 μg/m^3^ increase in PM_10_ increase on the seventh day prior. However, females were not sensitive to the concentration of PM_10_ on lag01 and lag1. The concentration of SO_2_ and CO in Nanchong did not affect the hospitalization of cardiovascular disease. Cumulative and single exposure to high levels of NO_2_ increased the risk of cardiovascular disease hospitalization. The risk increased by 6.26% (95% CI: 4.84–7.70%) for every 10 μg/m^3^ increase in the concentration of NO_2_ for lag07, and by 2.93% (95% CI: 1.90–4.02%). For all patients, O_3_ showed short-term cumulative effects (lag01 and lag02) and lag effects (lag1) on cardiovascular disease hospitalization. Female patients can be affected by the concentration of O_3_ in the previous 7 days, while males are not.

### 3.5 Subgroup analysis

**Figure 4:**
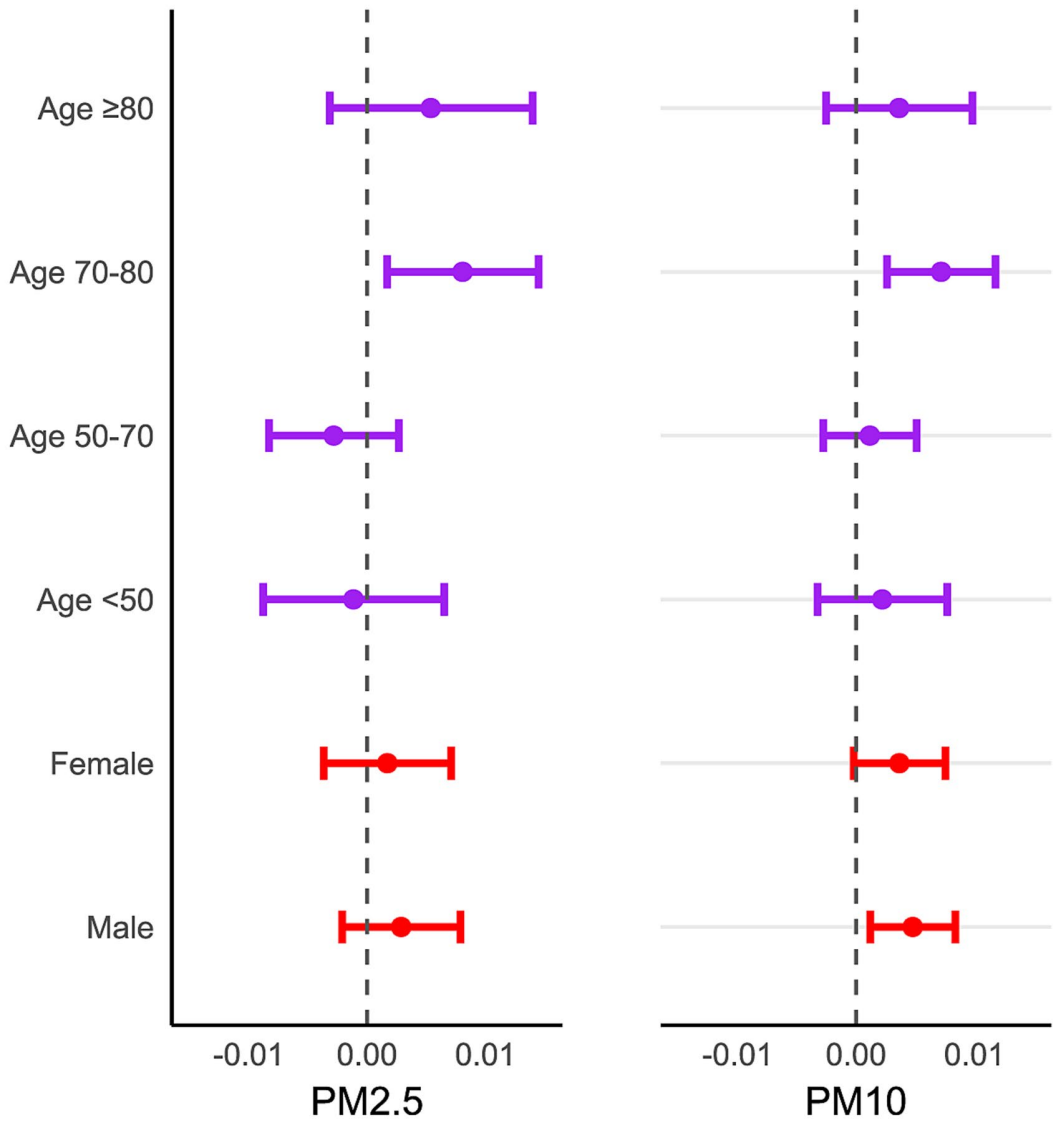
The excess risk (95% CI) of age and sex group in overall hospitalization for cardiovascular disease associated with a 10 μg/m^3^ increase in PM_2.5_ and PM_10_ on lag0 in Nanchong from June 1, 2015, to December 31, 2023.

To assess the influence of PM_2.5_ and PM_10_ on inpatients visits for CVD across different age and gender groups. The stratified analysis was performed based on sex and age at lag0 day. Figure 4 depicted that the risk of admission for CVD among male increased by 0.48% (95%CI:0.12–0.84%) for every 10 μg/m^3^ increase in PM_10_. However, we did not observe significant associations among female. In age-specific analysis, for CVD patients aged 70 to 80 years old, the risk of hospitalization increased by 0.81%(95%CI:0.17–1.50%) and 0.72%(95% CI: 0.26–1.18%) for every 10 μg/m^3^ increase in PM_2.5_ and PM_10_, respectively.

## 4 Discussion

This study represents the first attempt in Nanchong to investigate the association between air pollution and cardiovascular health. Generally, this study showed that short exposure to air pollution includes PM_10_, SO_2_, NO_2_ and O_3_ were associated with increased risk of admission among patients with cardiovascular disease. Furthermore, the effect of PM_10_ and SO_2_ on cardiovascular disease are varied by sex.

Particle matter has been adapted as a main indicator of air pollution worldwide. Particles pollutants PM_10_ and PM_2.5_ are named according to their aerodynamic diameters. The source of particles varies among different areas. For example, compared to developed counties, Pakistan suffered a higher PM_2.5_ concentration, in which also contain more heavy metal elements (13). Among the arid regions around the deserts such as Northern Africa, the Arabian Peninsula, Central Asia, the dust storms contribute significantly to local particle pollutant. Particles originating from dust storms typically contain mineral dust, whereas particulate matter emission by human activities is usually more complex and contains a higher proportion of organic and metal components (14, 15). However, due to the geographical location, air quality of Nanchong rarely affected by sandstorms. Therefore, the relationship between particles pollutant and wind speed in Nanchong is opposite with cities near sandstorms. In Nanchong, the wind can facilitate the dispersion of air pollutants, resulting in pollutant concentrations decreased in urban areas. Similar with most of the country, the concentration of particles matters peak during winter. This may be due to the dryness in winter promote the formation of particles matters, while adequate precipitation captures and remove aerosol particles from the atmosphere in summer (16). According to the update guidelines, WHO recommends a stricter criterion in which the recommendation daily concentration of PM_2.5_ was 15 μg/m^3^ and 45 μg/m^3^ for PM_10_ (17). Over the past 10 years in Nanchong, although the concentration of particles has continued to decrease, the average daily concentration of PM_2.5_ is 42.39 μg/m^3^, which remains significantly higher than the standard. PM_10_ and PM_2.5_ shown a great effect on human hearth due to that they can penetrate respiratory system and deposited in organ and tissues following the blood follow, especially in heart. Different from the other organs, heart is characterized as high energy demand and low repair capacity. On the one hand, PM disturb the oxidative respiratory chain and oxidation–reduction system, resulting in oxidative stress in heart. On the other hand, as a foreign substance, PM arouse inflammation response, however, inflammation response does not always clear they, resulting in continuous and excessive inflammation which promote heart disease. At the same time, particle matters also disturb the coagulation system and promote the aggravation of myocardial ischemia in coronary heart disease patients. A time-series analysis conducted in New York state also revealed that a 10 μg/m^3^ increment in PM_2.5_ concentrations contribute 1.37% increase in cardiovascular disease hospitalization (18). A prospective longitudinal cohort study conducted in Ohio found that exposure to PM_2.5_ increased risk of incident myocardial infarction (19). Another research conducted by Ma et al. (20) found that 10 μg/m^3^ increase on the concentration of PM_10_ was related with 14% increase in cardiovascular disease hospitalization on lag1. Furthermore, our results showed that PM_2.5_ has a stronger effect on cardiovascular disease compared to PM_10_. The greater impact of PM_2.5_ may be due to its smaller particles size, which results in a higher surface area. This make PM_2.5_ particles more likely to reach cardiovascular and exhibit higher reactivity ability (21, 22).

Since there are no volcanoes and heavy industry in Nanchong, the main source of SO_2_ and NO_2_ is the burning of fossil fuels, especially from transportation vehicles. WHO recommends 40 μg/m^3^ and 25 μg/m^3^of daily average concentrations for SO_2_ and NO_2_, respectively. In Nanchong, the daily average concentrations of SO_2_ and NO_2_ are lower than this standard. However, we also found that short exposure to SO_2_ and NO_2_ also can increase the number of hospitalizations count of cardiovascular disease patients. A study conducted in western China reported that cardiovascular admissions increased by 4.5%(95% CI 1–8.2%) for every 10 μg/m^3^ increase in SO_2_ in non-dust days (20). Mechanistic investigation revealed that SO_2_ induce mitochondrial and oxidation–reduction system through grapes transcriptome (23). Likewise, with pungent odor, SO_2_ will induce acute lung response and inflammation, which also can affect heart function through autonomic nervous system. Furthermore, most NO_2_ is quickly formed in the atmosphere from NO emissions by vehicles. A global assessment revealed that a 10 μg/m^3^ increase in NO_2_ concentration was associated 0.37% cardiovascular mortality among 398 cities (24). A Meta-analysis enrolled 204 time-series reported that a 10 μg/m^3^ increase of NO_2_ associated with 0.88% cardiovascular mortality (25). A prospective longitudinal observational cohort study conducted in England reported that NO_2_ promotes the development of cardiac remodeling and decrease cardiac pumping function (26). Moreover, NO_2_ has been implicated in promoting airway inflammation and increasing the airways’ vulnerability to viral infections (27). Among asthmatic children, NO_2_ increase the risk of respiratory viral infection and resulting in severity asthmatic (28, 29). This also contributes to the development of cardiovascular disease, especially for patients with previous heart disease.

Different from other air pollutant, O_3_ is not directly emitted by primary source. Nitrogen dioxide and volatile organic compounds works as a precursor in atmospheric and promote the formation of O_3_, in which UV radiation or thunderstorm activity is needed (30). Therefore, we can easily be found in Figure 2 that the concentration keeps a high concentration in summer and spring. The Ozone layer in atmosphere protects human from the UV radiation; however, excessive ground-level ozone is harmful to human health. Cumulative and lag effect also have been revealed in O_3_. The WHO recommended concentration of O_3_ is 60 μg/m^3^. In Nanchong, the average concentration of O_3_ is 69.63. μg/m^3^. A nationwide cohort conducted in China reported that per 10 μg/m^3^ increase in ozone concentrations related to 18.4% increase in cardiovascular disease mortality (31). As a strongly oxidative and highly reactive pollutant, O_3_ may induce coronary artery spasm and trigger acute episodes of ischemic heart disease (32). Furthermore, O_3_ also induce oxidative stress and inflammatory (33).

Consistent with previous studies, our results suggest that elder adults are more susceptible to air pollution, especially for 70–80 years old (34). Older adults are more likely suffer from chronic disease including CVD. Air pollution can trigger acute episode of CVD, leading to an increased need for urgent hospitalization. Furthermore, patients over the age of 80 usually have less outdoor activity and receive more care, which protect them from the hazards of air pollution. Compared to females, males show greater vulnerability to PM_10_, possibly related to that males are more likely to work outdoor, increasing their exposure to high level of particles matter. Additionally, our results reveal that the effect estimates for moving average lags were much higher than those for single day lags. This significantly indicated that the hazardous of air pollution to CVD exhibit a cumulative effect. It’s also underscores that efforts to reduce continuous exposure to air pollution yield substantial benefits.

In this study, we employed patients’ addresses from medical records to assess air pollution exposure, which may lead to exposure misclassification. Migrant workers who provided domicile addresses rather than actual residential addresses were excluded, potentially underestimating the impact of air pollution on cardiovascular disease. Additionally, factors such as traffic volume, mask-wearing, and mode of transportation were not fully accounted for, which could introduce bias. The potential impact of participant mobility within urban areas during the study period was also not addressed. While a single-pollutant model was used for simplicity, multiple-pollutant models are needed in future research to better capture real-world exposure. Finally, since the study was limited to Nanchong, caution should be exercised when generalizing the results to other regions.

## 5 Conclusion

Our study assesses the impact of air pollution on CVD patients in Nanchong, a region with a subtropical humid monsoon climate and mild air pollution. Our findings reveal short exposure to high concentrations of air pollution increases the risk of hospital admission for CVD. Efforts should be devoted to avoiding exposure to high and consistent levels of air pollution, especially for elder patients.

At the same time, medical institutions should prepare enough medical sources. Furthermore, patients aged 70–80 years old are more vulnerable to high air pollutant. These results suggest that older adult patients require additional care and further precautions to avoid exposure to high pollution levels. Policymakers need to develop policies to combat air pollution and ensure sufficient medical resources are available after severe air pollution days.

## Data Availability

The original contributions presented in the study are included in the article/Supplementary material, further inquiries can be directed to the corresponding authors.

## Footnotes

^1^ https://www.aqistudy.cn/historydata/
