# Supplementary material for: Associations between air pollution and hospitalization for cardiovascular disease: a time series study in Nanchong
Source: Front Public Health. 2025 Mar 7;13:1504411. doi: 10.3389/fpubh.2025.1504411 (PMC11925947; doi:10.3389/fpubh.2025.1504411)
Supplement: Supplementary file 1 [file Table_1.docx]

Supplement Table 1: Spearman Correlation Analysis of Local Meteorological Data and Air Pollutants in Nanchong City from 2015 to 2023.

|  | PM2.5  (μg/m^3^) | PM10  (μg/m^3^) | SO2  (μg/m^3^) | NO2  (μg/m^3^) | CO  (mg/m^3^) | O3  (μg/m^3^) | Daily Avg. Temperature  (℃) | Daily Max. Temperature  (℃) | Daily Min. Temperature  (℃) | Precipitation  (mm) | Wind Speed  (m/s) | Relative Humidity  (%) |
| --- | --- | --- | --- | --- | --- | --- | --- | --- | --- | --- | --- | --- |
| PM2.5 (μg/m^3^) | 1.000 |  |  |  |  |  |  |  |  |  |  |  |
| PM10 (μg/m^3^) | 0.963 | 1.000 |  |  |  |  |  |  |  |  |  |  |
| SO2 (μg/m^3^) | 0.442 | 0.445 | 1.000 |  |  |  |  |  |  |  |  |  |
| NO2(μg/m^3^) | 0.733 | 0.749 | 0.554 | 1.000 |  |  |  |  |  |  |  |  |
| CO(mg/m^3^) | 0.777 | 0.724 | 0.465 | 0.704 | 1.000 |  |  |  |  |  |  |  |
| O3 (μg/m^3^) | -0.130 | -0.037 | 0.113 | 0.016 | -0.127 | 1.000 |  |  |  |  |  |  |
| Daily Avg. Temperature (℃) | -0.352 | -0.295 | 0.074 | -0.271 | -0.336 | 0.592 | 1.000 |  |  |  |  |  |
| Daily Max. Temperature (℃) | -0.313 | -0.239 | 0.072 | -0.227 | -0.319 | 0.665 | 0.978 | 1.000 |  |  |  |  |
| Daily Min. Temperature (℃) | -0.392 | -0.355 | 0.066 | -0.318 | -0.349 | 0.491 | 0.977 | 0.920 | 1.000 |  |  |  |
| Precipitation (mm) | -0.399 | -0.462 | -0.073 | -0.313 | -0.222 | -0.160 | 0.004 | -0.074 | 0.112 | 1.000 |  |  |
| Wind Speed (m/s) | -0.346 | -0.311 | -0.217 | -0.376 | -0.260 | 0.209 | 0.088 | 0.087 | 0.098 | 0.250 | 1.000 |  |
| Relative Humidity(%) | -0.095 | -0.083 | -0.076 | -0.109 | -0.105 | 0.090 | 0.258 | 0.237 | 0.273 | -0.054 | -0.038 | 1.000 |
