# Supplementary material for: Associations between air pollution and hospitalization for cardiovascular disease: a time series study in Nanchong
Source: Front Public Health. 2025 Mar 7;13:1504411. doi: 10.3389/fpubh.2025.1504411 (PMC11925947; doi:10.3389/fpubh.2025.1504411)
Supplement: Supplementary file 2 [file Table_2.docx]

Supplement Table 2: Percentage (95% CI) in hospitalization for CVD associated with an increase of 10 units of pollutant at different lag days using single-pollutant models in Nanchong, 2014-2023.

| **Pollutant** | **Lag** | **RRI** | **95%CI_lower** | **95%CI_upper** | **Pollutant** | **Lag** | **RRI** | **95%CI_lower** | **95%CI_upper** |
| --- | --- | --- | --- | --- | --- | --- | --- | --- | --- |
| PM10 | lag07 | 1.18% | 0.75% | 1.61% | NO_2_ | lag07 | 6.26% | 4.84% | 7.70% |
|  | lag06 | 1.11% | 0.70% | 1.53% |  | lag06 | 5.92% | 4.56% | 7.30% |
|  | lag05 | 1.05% | 0.65% | 1.44% |  | lag05 | 5.58% | 4.27% | 6.90% |
|  | lag04 | 0.90% | 0.53% | 1.28% |  | lag04 | 5.14% | 3.89% | 6.41% |
|  | lag03 | 0.73% | 0.37% | 1.09% |  | lag03 | 4.73% | 3.53% | 5.94% |
|  | lag02 | 0.55% | 0.21% | 0.89% |  | lag02 | 4.54% | 3.40% | 5.69% |
|  | lag01 | 0.43% | 0.11% | 0.75% |  | lag01 | 4.29% | 3.21% | 5.38% |
|  | lag0 | 0.42% | 0.12% | 0.72% |  | lag0 | 4.20% | 3.18% | 5.22% |
|  | lag1 | 0.34% | 0.04% | 0.64% |  | lag1 | 3.31% | 2.31% | 4.32% |
|  | lag2 | 0.55% | 0.24% | 0.85% |  | lag2 | 3.17% | 2.17% | 4.18% |
|  | lag3 | 0.79% | 0.49% | 1.10% |  | lag3 | 2.81% | 1.81% | 3.83% |
|  | lag4 | 0.87% | 0.57% | 1.18% |  | lag4 | 3.36% | 2.35% | 4.38% |
|  | lag5 | 0.82% | 0.50% | 1.13% |  | lag5 | 3.40% | 2.38% | 4.42% |
|  | lag6 | 0.52% | 0.20% | 0.83% |  | lag6 | 2.99% | 1.96% | 4.02% |
|  | lag7 | 0.51% | 0.19% | 0.82% |  | lag7 | 2.93% | 1.90% | 3.98% |
| PM2.5 | lag07 | 1.16% | 0.55% | 1.76% | O_3_ | lag07 | 0.43% | -0.09% | 0.95% |
|  | lag06 | 1.12% | 0.54% | 1.70% |  | lag06 | 0.45% | -0.06% | 0.96% |
|  | lag05 | 1.05% | 0.50% | 1.60% |  | lag05 | 0.50% | 0.00% | 1.00% |
|  | lag04 | 0.88% | 0.36% | 1.41% |  | lag04 | 0.45% | -0.03% | 0.93% |
|  | lag03 | 0.68% | 0.18% | 1.18% |  | lag03 | 0.47% | 0.00% | 0.93% |
|  | lag02 | 0.45% | -0.02% | 0.92% |  | lag02 | 0.57% | 0.12% | 1.01% |
|  | lag01 | 0.25% | -0.19% | 0.69% |  | lag01 | 0.77% | 0.35% | 1.19% |
|  | lag0 | 0.23% | -0.19% | 0.65% |  | lag0 | 0.58% | 0.21% | 0.95% |
|  | lag1 | 0.21% | -0.21% | 0.63% |  | lag1 | 0.62% | 0.25% | 0.99% |
|  | lag2 | 0.63% | 0.21% | 1.06% |  | lag2 | -0.03% | -0.38% | 0.32% |
|  | lag3 | 0.90% | 0.47% | 1.33% |  | lag3 | 0.01% | -0.33% | 0.35% |
|  | lag4 | 0.96% | 0.53% | 1.40% |  | lag4 | 0.11% | -0.22% | 0.45% |
|  | lag5 | 0.92% | 0.48% | 1.36% |  | lag5 | 0.28% | -0.05% | 0.62% |
|  | lag6 | 0.52% | 0.08% | 0.97% |  | lag6 | 0.00% | -0.33% | 0.34% |
|  | lag7 | 0.39% | -0.06% | 0.84% |  | lag7 | 0.05% | -0.28% | 0.38% |
| SO_2_ | lag07 | -0.40% | -4.39% | 3.77% | CO | lag07 | -40.20% | -78.70% | 67.89% |
|  | lag06 | 0.00% | -3.90% | 4.05% |  | lag06 | -31.74% | -74.70% | 84.15% |
|  | lag05 | 0.24% | -3.54% | 4.16% |  | lag05 | -24.57% | -70.88% | 95.38% |
|  | lag04 | -0.05% | -3.69% | 3.73% |  | lag04 | -34.35% | -73.51% | 62.72% |
|  | lag03 | -0.27% | -3.75% | 3.34% |  | lag03 | -43.88% | -76.27% | 32.76% |
|  | lag02 | 0.61% | -2.73% | 4.06% |  | lag02 | -42.32% | -74.42% | 30.07% |
|  | lag01 | 0.22% | -2.88% | 3.41% |  | lag01 | -38.45% | -71.01% | 30.71% |
|  | lag0 | 0.45% | -2.28% | 3.25% |  | lag0 | -12.45% | -55.00% | 70.34% |
|  | lag1 | -0.11% | -2.79% | 2.64% |  | lag1 | -46.94% | -72.81% | 3.56% |
|  | lag2 | 0.84% | -1.84% | 3.59% |  | lag2 | -26.45% | -60.83% | 38.12% |
|  | lag3 | -1.82% | -4.46% | 0.91% |  | lag3 | -20.66% | -56.85% | 45.88% |
|  | lag4 | 0.50% | -2.20% | 3.27% |  | lag4 | 22.44% | -33.05% | 123.92% |
|  | lag5 | 0.81% | -1.88% | 3.57% |  | lag5 | 28.16% | -29.64% | 133.43% |
|  | lag6 | -0.75% | -3.43% | 2.01% |  | lag6 | -25.99% | -59.29% | 34.57% |
|  | lag7 | -1.40% | -4.08% | 1.36% |  | lag7 | -34.33% | -63.82% | 19.23% |
